# Activation of microglia induces symptoms of Parkinson’s disease in wild-type, but not in IL-1 knockout mice

**DOI:** 10.1186/1742-2094-10-143

**Published:** 2013-12-01

**Authors:** Sachiko Tanaka, Atsuko Ishii, Hirokazu Ohtaki, Seiji Shioda, Takemi Yoshida, Satoshi Numazawa

**Affiliations:** 1 Department of Pharmacology, Toxicology and Therapeutics, Division of Toxicology, School of Pharmacy, Showa University, 1-5-8 Hatanodai, Shinagawa-ku, Tokyo 142-8555, Japan; 2 Department of Anatomy, School of Medicine, Showa University, 1-5-8 Hatanodai, Shinagawa-ku, Tokyo 142-8555, Japan

**Keywords:** Microglia, IL-1β, Knockout mouse, Neuroinflammation, Parkinson’s disease

## Abstract

**Background:**

Parkinson’s disease (PD) is an age-related progressive neurodegenerative disorder caused by selective loss of dopaminergic neurons from the substantia nigra (SN) to the striatum. The initial factor that triggers neurodegeneration is unknown; however, inflammation has been demonstrated to be significantly involved in the progression of PD. The present study was designed to investigate the role of the pro-inflammatory cytokine interleukin-1 (IL-1) in the activation of microglia and the decline of motor function using IL-1 knockout (KO) mice.

**Methods:**

Lipopolysaccharide (LPS) was stereotaxically injected into the SN of mice brains as a single dose or a daily dose for 5 days (5 mg/2 ml/injection, bilaterally). Animal behavior was assessed with the rotarod test at 2 hr and 8, 15 and 22 days after the final LPS injection.

**Results:**

LPS treatment induced the activation of microglia, as demonstrated by production of IL-1β and tumor necrosis factor (TNF) α as well as a change in microglial morphology. The number of cells immunoreactive for 4-hydroxynonenal (4HNE) and nitrotyrosine (NT), which are markers for oxidative insults, increased in the SN, and impairment of motor function was observed after the subacute LPS treatment. Cell death and aggregation of α-synuclein were observed 21 and 30 days after the final LPS injection, respectively. Behavioral deficits were observed in wild-type and TNFα KO mice, but IL-1 KO mice behaved normally. Tyrosine hydroxylase (TH) gene expression was attenuated by LPS treatment in wild-type and TNFα KO mice but not in IL-1 KO mice.

**Conclusions:**

The subacute injection of LPS into the SN induces PD-like pathogenesis and symptoms in mice that mimic the progressive changes of PD including the aggregation of α-synuclein. LPS-induced dysfunction of motor performance was accompanied by the reduced gene expression of TH. These findings suggest that activation of microglia by LPS causes functional changes such as dopaminergic neuron attenuation in an IL-1-dependent manner, resulting in PD-like behavioral impairment.

## Background

Lipopolysaccharide (LPS) is a bacterial endotoxin known to stimulate immune responses [1]. In *in vivo* experiments, LPS activates the microglia, resulting in the release of inflammatory cytokines such as interleukin-1β (IL-1β) and tumor necrosis factor α (TNFα), which contribute to neurodegeneration [1-5]. Activation of the microglia has also been observed during the development of neurodegenerative conditions such as Alzheimer’s disease (AD), Parkinson’s disease (PD) and multiple sclerosis [6-8]. Several epidemiological studies have shown that non-steroidal anti-inflammatory drugs are associated with a reduced risk of developing PD [9-11]. These phenomena suggest that inflammation is implicated in neurodegenerative diseases [12]. PD is an age-related progressive neurodegenerative disorder caused by the selective loss of dopaminergic neurons from the substantia nigra (SN) to the striatum [13]. However, the initial factor that triggers neurodegeneration is unknown. PD animal models have been created by exposing animals to chemical toxins such as 1-methyl 4-phenyl 1,2,3,6,-tetrahydropyridine (MPTP) [14-16] and 6-hydroxydopamine (6-OHDA) [17]. These toxins selectively modulate dopaminergic neurons via the uptake by the dopamine transporter. However, these animal models do not encompass all the prevailing pathologies of PD. Therefore, we designed a PD animal model where there is neuroinflammation in the mouse brain. In our previous studies, we demonstrated that subacute administration of LPS (20 μg/2 μL/injection, daily, bilaterally for 5 consecutive days) into the CA1 region of the rat and mouse hippocampus activated the microglia and increased production of IL-1β and TNFα, concomitantly resulting in learning and memory deficits in the animals as assessed using a step-through passive avoidance test [5,18]. These results suggest that inflammation affects neuronal function. Furthermore, IL-1β plays an important role in LPS-induced impairment of learning and memory using IL-1α/β knockout (KO) mice [18]. In the present study, we modified the regimen and obtained evidence that suggests there are PD-like pathological changes and symptoms in the animal model. In addition, LPS-induced microglial activation causes toxicity in dopaminergic neurons in an IL-1-dependent manner. The results of the present study may lead to a better understanding of the roles of IL-1 in the activation of the microglia and the mechanisms underlying neurodegenerative diseases.

## Methods

### Materials

The following reagents were obtained from commercial sources: LPS (from *Escherichia coli* serotype 055:B5; L2880, endotoxin level 3000000 EU/mg), monoclonal anti-mouse glial fibrillary acidic protein (GFAP) antibody from Sigma-Aldrich (St Louis, MO), monoclonal goat anti-mouse CD11b antibody from Serotec Ltd (Oxford, UK), goat anti-murine IL-1β antibody from R&D Systems (Minneapolis, MN), polyclonal rabbit anti-Iba1 antibody from Wako Pure Chemical Industries (Osaka, Japan), polyclonal anti-3-nitrotyrosine antibody, Alexa Fluor 546 donkey anti-goat IgG antibody, Alexa Fluor 488 goat anti-rat IgG antibody and Alexa Fluor 488 goat anti-mouse IgG antibody from Molecular Probes (Eugene, OR), monoclonal anti-α-synuclein antibody from Santa Cruz Biotechnology, Inc (Dallas, TX), polyclonal anti-4-hydroxynonenal (4HNE) antibody from ENZO Life Sciences, Inc (Famingdale, NY) and anti-nitrotyrosine (NT) antibody from Merck (Darmstadt, Germany). All other chemicals were of analytical or the highest grade commercially available.

### Animals, surgical operations and LPS treatment

All animal experiments were conducted in accordance with the Showa University Animal Experiment and Welfare Regulations. The IL-1 KO male mice, carrying a null mutation in both the IL-1α and IL-1β genes, and the TNFα Κ Ο male mice were established by Horai *et al*. [19] and Tagawa *et al*. [20], respectively. Male BALB/c mice were purchased from Sankyo Laboratory Service (Tokyo, Japan). The mice (at 9 to 10 weeks old) were anesthetized with sodium pentobarbital (50 mg/kg intraperitoneally) and immobilized in a stereotaxic frame. Two guide cannulas were implanted and used to inject LPS into both sides of the substantia nigra pars reticulata (SNR) (2.92 mm posterior, 1.25 mm lateral and 4.95 mm ventral to the bregma). These cannulas were fixed to the skull with dental cement. Eight days after surgery, the mice were injected bilaterally with either LPS (5 μg) dissolved in 2 μL of phosphate-buffered saline (PBS) or PBS alone under isoflurane anesthesia. Acute treatment with PBS or LPS (5 μg/2 μL/injection, bilaterally) was carried out to study the immunohistochemistry of the microglia. Subacute PBS or LPS injections (5 μg/2 μL/injection, bilaterally) were administered daily for 5 consecutive days under isoflurane anesthesia. These animals were used for behavioral tests, gene expression experiments and to study the immunohistochemistry of the brain section.

### Behavioral analysis: the rotarod test

Motor coordination was assessed with a rotating rod apparatus (Panlab Harvard Apparatus, Barcelona, Spain). The rod was 3 cm in diameter. The mice were placed on the rod when it was rotating at 4 rpm. The rotation speed was increased from 4 to 40 rpm within 5 min. The latency was recorded for each animal. This is the time (in seconds) before they fall. Each mouse was tested three times and the median time of the three trials was calculated. The results are expressed as the mean ± standard error of the mean (SEM) in each group.

### Immunohistochemistry

Six hours after acute LPS or PBS treatment, the mice were anesthetized with pentobarbital and were perfused transcardially with saline followed by 0.1 M phosphate buffer (PB, pH 7.2) containing 2% paraformaldehyde [21]. The brains were removed and immersed for 1 day in 0.1 M PB containing 2% paraformaldehyde and then for 2 days in 0.1 M PB containing 20% sucrose. The brains were then embedded in a mixture of 20% sucrose in 0.1 M PB and Tissue Teck (2:1; Miles Inc, Elkhart, IN), frozen on dry ice, and stored at -80°C until studied. Subsequently, 10 μm sections were cut with a cryostat and mounted onto gelatin-coated slides.

For staining of IL-1β, CD11b and tyrosine hydroxylase (TH), sections were pre-incubated in 5% normal horse serum. The sections were incubated with goat anti-mouse IL-1β antibody (1:100) and then were rinsed and incubated with Alexa 546-labeled donkey anti-goat IgG antibody (1:400). After that, they were incubated with rat anti-mouse CD11b antibody (1:100) followed by labeling with Alexa 488-labeled goat anti-rat IgG antibody (1:400). Then they were incubated with polyclonal anti-TH antibody (1:1000) followed by labeling with Alexa 350-labeled goat anti-rabbit IgG antibody (1:400).

For staining of 4HNE and NT, following pre-incubation in 5% normal horse serum sections were incubated with rabbit anti-4HNE antibody (1:500) or rabbit anti-NT antibody (1:500) followed by labeling with Alexa 546-labeled goat anti-rabbit IgG antibody (1:400) and then incubated with 4',6-diamidino-2-phenylindole (DAPI) solution (1:10000) to stain the nuclei.

For staining of α-synuclein, following pre-incubation in 5% normal goat serum, sections were incubated with monoclonal anti-α-synuclein antibody(1:500) followed by labeling with Alexa 546-labeled goat anti-mouse IgG antibody (1:400). Then they were incubated with polyclonal anti-TH antibody (1:1000), followed by Alexa 488-labeled goat anti-rabbit IgG antibody (1:400) and DAPI solution. Labeling was imaged with a fluorescence microscope (Olympus AX-70; Olympus, Tokyo, Japan).

For TH immunostaining using diaminobenzidine, the mice were sacrificed 6 hr after the final injection of the subacute treatment. Slice was prepared as presented above. Slice sections were pre-incubated in 5% normal goat serum after endogenous peroxidase blocking by 0.1 M PB containing 0.3% H_2_O_2_ and incubated with polyclonal anti-TH antibody (1:1000). The sections were rinsed and incubated with biotinylated horse anti-rabbit IgG (1:1000). Then they were incubated in an avidin-biotin complex solution followed by diaminobenzidine (DAB kit, Vector, Burlingame, CA).

Fluoro-Jade B staining was used to detect neurodegenerative cells [22]. Sections were fixed with 4% paraformaldehyde solution for 20 min. After washing twice with PBS and once with water, the sections were immersed in 0.06% KMnO_4_ for 15 min and then rinsed three times with purified water. Fluoro-Jade B solution (0.01% Fluoro-Jade B: 0.1% acetic acid = 1:19) was applied for 30 min at room temperature and then rinsed off with purified water, four times. The sections were dried with cold air and immersed three times in xylene for 2 min. One drop of marinol was put on top and a cover was placed on them. Labeling was imaged with a fluorescence microscope (Olympus AX-70; Olympus, Tokyo, Japan).

### Gene expression

The mice were decapitated 6 hr after the acute PBS or LPS injection or 6 hr after the final PBS or LPS injection of the subacute treatment. The midbrains were dissected according to the method of Glowinski and Iversen [23] and stored at -80°C until use. Total RNA was extracted using the QIAGEN RNeasy Lipid Tissue Mini kit (QIAGEN, Hilden, Germany). Real-time RT-PCR was carried out using QuantiTect SYBR Green RT-PCR system (QIAGEN). Primers for IL-1β, TNFα, TH and glyceraldehyde 3-phosphate dehydrogenase (GAPDH) were prepared by QuantiTect Primer Assays (QIAGEN). The amplification conditions were 40 cycles at 94°C for 15 s, 55°C for 30 s and 72°C for 30 s. Quantitative data were obtained from the relative standard curve. mRNA expression was normalized using GAPDH as an endogenous control.

### Statistical analysis

Immunostaining in the substantia nigra pars compact (SNC) was quantified by counting positively stained cells. The number of positively stained cells in four brain sections was counted and averaged (cells/mm^2^). The statistical analysis used the Mann–Whitney test for the immunostaining, behavioral and gene expression data.

## Results

### Activation of the microglia after lipopolysaccharide treatment

**Figure 1:**
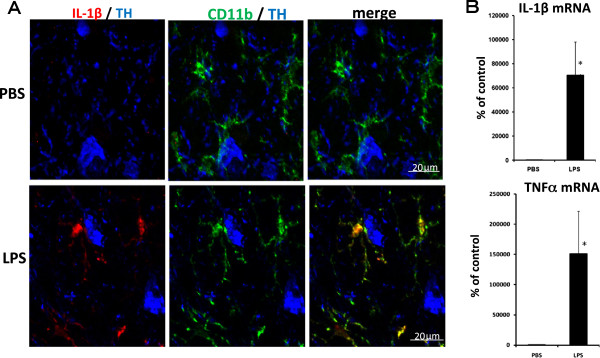
**Expression of inflammatory cytokine in an acute LPS-treated mouse. (A)** Immunohistochemical analysis of the effect of LPS treatment on the microglia in the SNC. PBS (2 μL/injection) or LPS (5 μg/2 μL/injection) was injected into the SNR bilaterally. These representative photomicrographs are coronal sections of the SNC at 6 hr after PBS (upper row) or acute LPS treatment (lower row). Triple immunofluorescence staining was performed with CD11b (green), IL-1β (red) and TH (blue) antibodies. Immunoreactivity for IL-1β was co-localized with that for CD11b after LPS treatment (yellow in the merged photomicrograph). Scale bars: 20 μm **(B)** Effect of LPS treatment on the gene expression of inflammatory cytokine in the midbrain. The midbrain was obtained 6 hr after the acute LPS treatment. Values are expressed as percentages of those in the PBS-treated group and represented as mean ± SEM. *P < 0.05 compared with the PBS-treated group. IL, interleukin; LPS, lipopolysaccharide; PBS, phosphate-buffered saline; SEM, standard error of the mean; SNC, substantia nigra pars compact; SNR, substantia nigra pars reticulata; Τ Η , tyrosine hydroxylase.

An experiment was conducted to determine whether the microglia was activated by acute LPS treatment (5 μg/2 μL/injection). There were many TH antibody-labeled dopaminergic neurons in the SNC. Immunohistochemical analysis revealed no IL-1β immunoreactivity in the SNC from mice treated with PBS (Figure 1A). In contrast, cells with strong immunoreactivity for IL-1β were observed 6 hr after the LPS injection. To identify the cells expressing IL-1β, a triple-label immunohistochemical study was performed for IL-1β, CD11b and TH. IL-1β immunopositive cells were co-localized with cells immunoreactive to CD11b. In the early stage of activation, LPS induced an increase in IL-1β signals in the microglia, the shape of which remained in the ramified form 6 hr after the acute LPS treatment. The gene expressions of IL-1β and TNFα in the midbrain were still noticeably higher 6 hr after the acute LPS treatment (Figure 1B).

**Figure 2:**
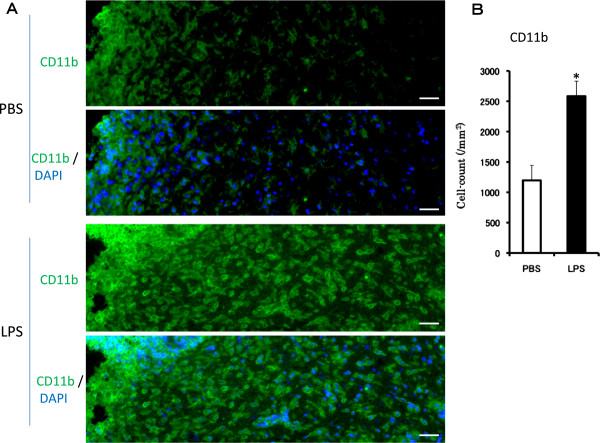
**Immunohistochemical analysis of the effect of the subacute LPS treatment on CD11b-positive microglia.** PBS (2 μL/injection, bilaterally) or LPS (5 μg/2 μL/injection, bilaterally) was injected into the SNR daily for 5 consecutive days. **(A)** Representative photomicrographs are coronal sections of the SNC 6 hr after the final PBS (upper rows) or LPS treatment (lower rows). Double immunofluorescence staining was performed with a CD11b antibody (green) and DAPI (blue). Scale bars: 50 μm **(B)** Number of CD11b immunopositive cells in the SNC. The number of microglial cells was counted between 200 μm and 300 μm from the injection site. The results are expressed as the mean ± SEM for four mice in each group. DAPI, 4',6-diamidino-2-phenylindole; IL, interleukin; LPS, lipopolysaccharide; PBS, phosphate-buffered saline; SEM, standard error of the mean; SNC, substantia nigra pars compact; SNR, substantia nigra pars reticulata.

We demonstrated the long-term activation of the microglia after the subacute treatment with LPS for 5 days (Figure 2). In the SN of mice injected with PBS, the majority of the CD11b immunopositive cells exhibited a resting or ramified state (Figure 2A, upper rows). After the subacute LPS treatment, the CD11b-immunopositive cells were activated, as indicated by an increased number of cells (Figure 2B) as well as a change in their morphology to a round and blunt shape (amoeboid form, Figure 2A, lower rows). The CD11b-immunopositive cells had an activated phenotype. These results suggest that the subacute LPS treatment caused a sustained activation of the microglia, resulting in an increase in the number of microglial cells and morphological changes.

### Detection of oxidative stress

**Figure 3:**
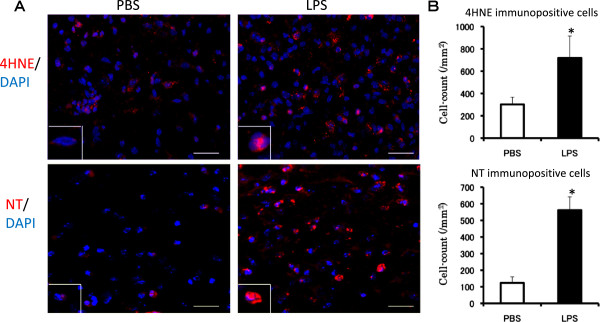
**Immunohistochemical analysis of the effect of subacute LPS treatment on oxidative stress markers.** PBS (2 μL/injection, bilaterally) or LPS (5 μg/2 μL/injection, bilaterally) was injected into the SNR daily for 5 consecutive days. **(A**) Representative photomicrographs are coronal sections of the SNC region 6 hr after the final PBS (upper row) or LPS treatment (lower row). Double immunofluorescence staining was performed with 4HNE (red, upper row) or NT (red, lower row) antibody and DAPI (blue). Scale bars: 20 μm **(B)** Numbers of 4HNE and NT immunopositive cells in the SNC. The results are expressed as the mean ± SEM for four mice in each group. 4HNE, 4-hydroxynonenal; DAPI, 4',6-diamidino-2-phenylindole; LPS, lipopolysaccharide; PBS, phosphate-buffered saline; SEM, standard error of the mean; SNC, substantia nigra pars compact; SNR, substantia nigra pars reticulata.

4HNE is a product of lipid peroxidation and its increased production is a biomarker of oxidative stress. The number of 4HNE-positive cells in the SNC was significantly increased 6 hr after the final LPS injection (Figure 3A,B, upper row). NT is an indicator of cell damage, the activation of inflammation and NO production [39]. NT-immunopositive cells were seen to increase up to fourfold 6 hr after the final LPS injection compared with the PBS-treated group (Figure 3A,B, lower row). These results suggest that oxidative stress was induced in the SNC by the subacute LPS treatment.

### Detection of cell death using Fluoro-Jade B and tyrosine hydroxylase immunostaining

**Figure 4:**
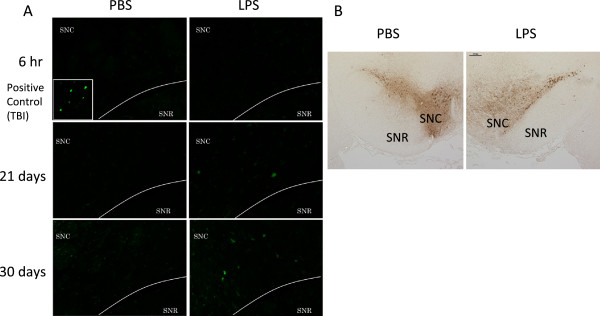
**Detection of cell death using Fluoro-Jade B and TH immunostaining.** PBS (2 μL/injection, bilaterally) or LPS (5 μg/2 μL/injection, bilaterally) was injected into the SNR daily for 5 consecutive days. **(A)** The representative photomicrographs of Fluoro-Jade B staining are coronal sections of the SNC 6 hr, 21 days and 30 days after the final PBS (left) or LPS treatment (right). The positive control was prepared after a traumatic brain injury (TBI) in the cortex. **(B)** The representative photographs of TH immunostaining using diaminobenzidine, are coronal sections of the SN 6 hr after the final PBS (left) or LPS treatment (right). LPS, lipopolysaccharide; PBS, phosphate-buffered saline; SN, substantia nigra; SNC, substantia nigra pars compact; SNR, substantia nigra pars reticulata; Τ Η , tyrosine hydroxylase.

Cell death was seen in coronal sections of the SN using Fluoro-Jade B staining (Figure 4A). Six hr after the final injection, degenerating neurons stained with Fluoro-Jade B were not observed. However, 21 and 30 days after the final injection, Fluoro-Jade B-positive cells were detected in a similar manner as observed in the positive control specimens prepared after a traumatic cortical brain injury. Furthermore, the number of TH antibody-labeled dopaminergic neurons in the SNC decreased 6 hr after the final LPS injection (Figure 4B). These results indicate that delayed cell death was induced in the SNC by the subacute LPS treatment.

### α-synuclein expression in lipopolysaccharide-treated mice

**Figure 5:**
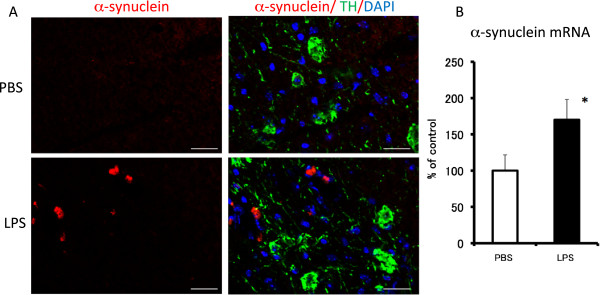
**Immunohistochemical analysis of the effect of subacute LPS treatment on α-synuclein.** PBS (2 μL/injection, bilaterally) or LPS (5 μg/2 μL/injection, bilaterally) was injected into the SNR daily for 5 consecutive days. **(A)** Representative photomicrographs are coronal sections of the SNC 30 days after the final PBS (upper) or LPS treatment (lower). Triple immunofluorescence staining was performed with α-synuclein (red) antibody, TH (green) antibody and DAPI (blue). Scale bars: 20 μm **(B)** Effect of LPS treatment on the gene expression of α-synuclein in the midbrain 30 days after the final LPS treatment. Values are expressed as percentages of those in the PBS-treated group and represented as mean ± SEM. *P < 0.05 compared with the PBS-treated group. DAPI, 4',6-diamidino-2-phenylindole; LPS, lipopolysaccharide; PBS, phosphate-buffered saline; SEM, standard error of the mean; SNC, substantia nigra pars compact; SNR, substantia nigra pars reticulata; Τ Η , tyrosine hydroxylase.

We performed an immunohistochemical analysis to see if there was any α-synuclein protein in the SNC (Figure 5). α-synuclein-immunopositive cells were observed 30 days after the final LPS injection (Figure 5A). α-synuclein gene expression in the midbrain area was unchanged 6 hr after the final LPS treatment compared with its expression in the PBS-treated group (data not shown). However, 30 days after the final LPS injection, α-synuclein gene expression was significantly increased to 170% of that for the PBS-treated group (Figure 5B).

### Assessment of animal behavior using the rotarod test

**Figure 6:**
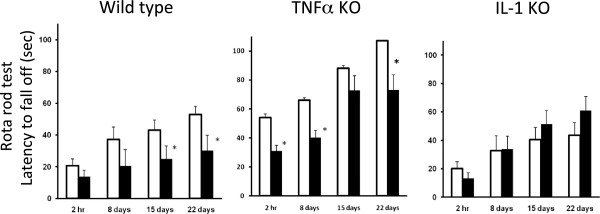
**Effect of subacute LPS treatment on performance in the rotarod test for wild-type, IL-1 KO and TNFα KO mice.** PBS (2 μL/injection, bilaterally, open column) or LPS (5 μg/2 μL/injection, bilaterally, closed column) was injected into the SNR daily for 5 consecutive days. The rotarod test was conducted at 2 hr and 8, 15 and 22 days after the final PBS or LPS treatment. The test was performed three times and latency to fall off was recorded for each trial. The median latency time was calculated for each mouse. The results are expressed as the mean ± SEM in each group. *P < 0.05 compared with the PBS-treated group. IL, interleukin; KO, knockout; LPS, lipopolysaccharide; PBS, phosphate-buffered saline; SEM, standard error of the mean; TNF, tumor necrosis factor.

Behavioral tests were performed 2 hr and 8, 15 and 22 days after the final LPS injection. The latency of the mice increased during the trial (Figure 6). In wild-type mice, LPS produced a lower latency to fall off, which was significant 15 days and 22 days after the final injection. These results suggest that the LPS treatment resulted in behavioral impairment of the motor function. We used genetically modified animals to determine whether the pro-inflammatory cytokine was involved in the LPS-induced behavioral deficit. The behavioral tests were performed with the IL-1α/β double KO and TNFα KO mice treated either with PBS or LPS. The latency of the IL-1 KO and TNFα KO mice also increased during the trial, like the wild-type mice (Figure 6). TNFα KO mice treated with LPS had a significantly lower latency to fall off 2 hr, 8 days and 22 days after the final LPS injection. However, there was no difference between the PBS and LPS treatments for the IL-1 KO mice. These results suggest that the LPS treatment caused impairment of the motor function in the wild-type and TNFα KO mice, but not in the IL-1 KO mice.

### Tyrosine hydroxylase gene expression in wild-type, TNFα and IL-1 KO mice

**Figure 7:**
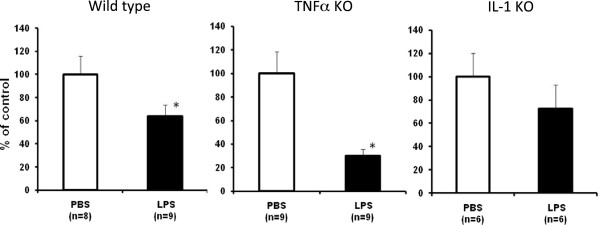
**Effect of subacute LPS treatment on the gene expression of tyrosine hydroxylase in the midbrain.** PBS (2 μL/injection, bilaterally) or LPS (5 μg/2 μL/injection, bilaterally) was injected into the SNR daily for 5 consecutive days. The midbrain was obtained 6 hr after the final PBS or LPS treatment. Values are expressed as percentages of those in the PBS-treated group and represented as mean ± SEM. *P < 0.05 compared with the PBS-treated group. IL, interleukin; KO, knockout; LPS, lipopolysaccharide; PBS, phosphate-buffered saline; SEM, standard error of the mean; SNR, substantia nigra pars reticulata; TNF, tumor necrosis factor.

We analyzed the gene expression of TH, a marker for dopaminergic and noradrenergic neurons, in the wild-type, TNFα and IL-1 KO mice 6 hr after the final LPS injection. The subacute LPS treatment significantly suppressed TH gene expression in the wild-type and TNFα KO mice, but not IL-1 KO mice (Figure 7). These results for TH expression are in agreement with those obtained from the behavior tests.

## Discussion

In the present study, we investigated whether *in vivo* activation of the microglia leads to impaired neuronal function and found that LPS-induced functional outcomes in terms of decreased motor performance correlated well with changes in immunohistochemistry and gene expression.

PD models have been created by exposing animals to toxins such as MPTP [14-16] and 6-OHDA [17]. These toxins have been demonstrated to cause PD-like symptoms with massive losses of dopaminergic neurons in the SN. These toxins are selectively taken up by dopaminergic neurons via the dopamine transporter and cause neuronal cell death without α-synuclein aggregation. In contrast, neuronal cell death is not believed to be the first event in the development of PD. What triggers PD in humans has not been determined. Therefore, these toxins do not induce changes that completely mimic PD-like pathology.

We chose LPS to induce microglial activation and neuronal inflammation in the SN of mice. In the LPS-induced inflammation model, dopaminergic neurons were not directly injured by the endotoxin, which may cause deterioration of neurons via the microglia. The present study indicated that LPS treatment caused the activation of the microglial cells that produce IL-1β and TNFα as well as changes in microglial morphology. These findings are comparable to those previously reported for our rat and mouse models [5,18], in which LPS injected into the hippocampus induced learning and memory deficits. TNFα and IL-1β are elevated in patients with PD [24-26] and animal models [27,28]. The activated microglia increases the secretion of pro-inflammatory cytokines and activates pro-oxidant enzymes such as nicotinamide adenine dinucleotide phosphate oxidase and inducible nitric oxide synthase (iNOS), resulting in the production of reactive oxygen species (ROS) and nitric oxide [29,30].

Oxidative stress contributes to the neurodegenerative process in AD and PD [31,32]. PD is characterized by the selective loss of dopaminergic neurons in the SN, and dopamine has been suggested to be the main endogenous toxin causing oxidative stress in the genesis of PD [33]. Dopaminergic neurons are vulnerable to oxidative stress. Dopamine is easily oxidized by molecular oxygen to produce ROS such as superoxide anions and hydrogen peroxide and forms aminochrome via dopamine *o*-quinone [34]. Iron accumulates in the SN of patients with PD [35]. Auto-oxidation of dopamine is stimulated by iron in patients with PD, which causes the degeneration of dopaminergic neurons by enhancing oxidative stress [34]. Levels of free and protein-bound 4HNE, a toxic aldehyde produced by the peroxidation of fatty acids, were significantly elevated in the ventricular fluid of patients with AD [36] and a PD animal model [37]. 4HNE induces apoptosis with caspase activation and DNA fragmentation [38]. In our LPS-induced animal model, we detected an increase in the number of cells immunopositive for 4HNE, which could be involved in neuronal cell death. We also observed that LPS increased the number of cells immunopositive for NT, which has been identified as an indicator of cell damage and inflammation as a result of the production of NO and oxidative stress [39]. LPS stimulates the induction of iNOS and its product NO reacts with superoxide to produce peroxynitrite and eventually nitration of proteins, which might contribute to disturbing protein functions resulting in the pathological processes seen during neurodegeneration.

α-synuclein is a major component of Lewy bodies, a pathological hallmark of PD, and is involved in neurodegeneration [40]. It has been reported that LPS stimulates the production of α-synuclein in the SN of rats [41]. The present study demonstrated that there was α-synuclein gene expression and cell death 22 and 30 days after the final LPS injection, respectively. However, the changes in animal behavior started just after the final LPS injection, suggesting that impairment of the motor function appears before cell death. These results suggest that the subacute injection of LPS into the SNR induces PD-like pathogenesis and symptoms in mice, which mimic the progressive changes of PD including the aggregation of α-synuclein that causes the dysfunction of motor performance. Therefore, a subacute LPS treatment could be a novel regimen to create animal models of PD.

We did not determine the type of cells that took part in the delayed cell death in this study. It is well known that 80% of neurons in the SNC are dopaminergic. Numbers of TH-immunopositive cells decreased 6 hr after the LPS final injection. Therefore, it is suggested that Fluoro-Jade B-stained cells are dopaminergic neurons in the SNC.

Several reports attest the involvement of IL-1 in neurodegenerative diseases. Increased iNOS immunoreactivity, which is normally observed after brain ischemia, is diminished in IL-1 KO mice. These mice exhibit markedly reduced neuronal loss and apoptotic cell death when they experience a transient cardiac arrest [42]. In a spinal cord injury model, the size of the lesion area decreased in IL-1 KO mice compared with wild-type mice [43]. We previously demonstrated that LPS-induced deficits of learning and memory did not occur for IL-1 KO mice [18]. These findings indicate that IL-1 plays an important role in neurodegenerative disorders in which a neuronal dysfunction is associated with the activation of the microglia and increased IL-1 expression.

The present study demonstrated that behavioral deficits do not occur following a subacute LPS treatment in IL-1 KO mice. TH gene expression, the late-limiting enzyme for dopamine synthesis, was attenuated by the LPS treatment in wild-type and TNFα KO mice, but not in IL-1 Κ Ο mice. Impairment of the motor function was accompanied by an alteration of TH gene expression. It appears that increased IL-1 expression in the early stage of PD may trigger microglial activation and may be involved in neurodegeneration. Thus, neuroinflammation including an increase of IL-1 levels and augmentation of its signaling pathway may contribute to the dopaminergic dysfunction. If we had checked dopamine levels, we may have found direct evidence for dopaminergic neuron attenuation and motor dysfunction. We will check dopamine levels in the following study. Our present results suggest that IL-1 plays an important role in mediating LPS-induced functional changes of motor performance. However, the precise mechanism by which microglial activation occurs remains to be determined.

## Conclusions

The findings presented in this study provide evidence that activation of the microglia by LPS causes functional changes such as dopaminergic neuron attenuation in an IL-1-dependent manner, resulting in PD-like behavioral impairment. The LPS-induced animal model for PD could be a useful tool for clarifying mechanisms underlying the neurodegenerative disease and, therefore, should be evaluated further.

## Abbreviations

4HNE: 4-hydroxynonenal; 6-OHDA: 6-hydroxydioamine; AD: Alzheimer’s disease; DAPI: 4',6-diamidino-2-phenylindole; GAPDH: Glyceraldehyde 3-phosphate dehydrogenase; GFAP: Glial fibrillary acidic protein; IL: Interleukin; iNOS: inducible nitric oxide synthase; KO: Knockout; LPS: Lipopolysaccharide; MPTP: 1-methyl 4-phenyl 1,2,3,6,-tetrahydropyridine; NT: Nitrotyrosine; PB: Phosphate buffer; PBS: Phosphate-buffered saline; PD: Parkinson’s disease; ROS: Reactive oxygen species; RT-PCR: Real-time polymerase chain reaction; SEM: Standard error of the mean; SN: Substantia nigra; SNC: Substantia nigra pars compact; SNR: Substantia nigra pars reticulata; Τ Η : Tyrosine hydroxylase; TNF: Tumor necrosis factor.

## Competing interests

The authors declare that they have no competing interests.

## Authors’ contributions

ST designed the study, established the protocols and drafted the manuscript. AI carried out the experiments and the statistical analysis. HO helped to establish the protocols and participated in the data analysis. SS and TY participated in the data analysis and helped to draft the manuscript. SN coordinated the experiments and co-wrote the manuscript. All authors read and approved the final manuscript.

## References

[B1] Quan N Sundar SK Weiss JM Induction of interleukin-1 in various brain regions after peripheral and central injections of lipopolysaccharide J Neuroimmunol 1994 49 125 134 10.1016/0165-5728(94)90188-0 8294551

[B2] Gayle DA Ling Z Tong C Landers T Lipton JW Carvey PM Lipopolysaccharide (LPS)-induced dopamine cell loss in culture: roles of tumor necrosis factor-α, interleukin-1β, and nitric oxide Brain Res Dev 2002 133 27 35 10.1016/S0165-3806(01)00315-7 11850061

[B3] Gasparini L Ongini E Maucci R Rosi S Ronchetti D Wenk G McGann K Hauss-Wegrzyniak B Attenuation of chronic neuroinflammation by a nitric oxide releasing derivative of the antioxidant ferulic acid J Neurochem 2004 89 484 493 10.1111/j.1471-4159.2004.02359.x 15056291

[B4] Fogal B Hewett JA Hewett SJ Interleukin-1β potentiates neuronal injury in a variety of injury models involving energy deprivation J Neuroimmunol 2005 161 93 100 10.1016/j.jneuroim.2004.12.007 15748948

[B5] Tanaka S Ide M Shibutani T Ohtaki H Numazawa S Shioda S Yoshida T Lipopolysaccharide-induced microglial activation induces learning and memory deficits without neuronal cell death in rats J Neurosci Res 2006 83 557 566 10.1002/jnr.20752 16429444

[B6] McGeer PL Itagaki S Boyes BE McGeer EG Reactive microglia are positive for HLA-DR in the substantia nigra of Parkinson’s and Alzheimer’s disease brains Neurology 1988 38 1285 1291 10.1212/WNL.38.8.1285 3399080

[B7] Dickson DW Lee SC Mattiace LA Yen SH Brosnan C Microglia and cytokines in neurological disease, with special reference to AIDS and Alzheimer’s disease Glia 1993 7 75 83 10.1002/glia.440070113 8423065

[B8] Bo L Monk S Kong PA Nyland H Pardo CA Trapp BD Detection of MHC class II antigens on macrophages and microglia, but not on astrocytes and endothelia in active multiple sclerosis lesions J Neuroimmunol 1994 51 135 146 10.1016/0165-5728(94)90075-2 8182113

[B9] Chen H Jacobs E Schwarzschild MA McCullough ML Calle EE Thun MJ Ascherio A Nonsteroidal antiinflammatory drug use and the risk for Parkinson’s disease Ann Neurol 2005 58 963 967 10.1002/ana.20682 16240369

[B10] Wahner AD Bronstein JM Bordelon YM Ritz B Nonsteroidal anti-inflammatory drugs may protect against Parkinson disease Neurology 2007 69 1836 1842 10.1212/01.wnl.0000279519.99344.ad 17984451

[B11] Rees K Stowe R Patel S Ives N Breen K Clarke CE Ben-Shlomo Y Non-steroidal anti-inflammatory drugs as disease-modifying agents for Parkinson’s disease: evidence from observational studies Cochrane Database Syst Rev 2011 11 CD008454 22071848 10.1002/14651858.CD008454.pub2 PMC13579550

[B12] Lindvall O Ekdahl CT Claasen JH Bonde S Kokaia Z Lindvall O Inflammation is detrimental for neurogenesis in adult brain Proc Natl Acad Sci USA 2003 100 13632 13637 10.1073/pnas.2234031100 14581618 PMC263865

[B13] Olanow CW Tatton WG Etiology and pathogenesis of Parkinson’s disease Annu Rev Neurosci 1999 22 123 144 10.1146/annurev.neuro.22.1.123 10202534

[B14] Heikkila RE Hess A Duvoisin RC Dopaminergic neurotoxicity of 1-methyl-4-phenyl-1,2,5,6-tetrahydropyridine in mice Science 1984 224 1451 1453 10.1126/science.6610213 6610213

[B15] Dauter W Przedborski S Parkinson’s disease: mechanisms and models Neuron 2003 39 889 909 10.1016/S0896-6273(03)00568-3 12971891

[B16] Fornai F Schluter OM Lenzi P Gesi M Ruffoli R Ferrucci M Lazzeri G Busceti CL Pontarelli F Battaglia G Pellegrini A Nicoletti F Ruggieri S Paparelli A Sudhof TC Parkinson-like syndrome induced by continuous MPTP infusion: convergent roles of the ubiquitin-proteasome system and **α**-synuclein Proc Natl Acad Sci USA 2005 102 3413 3418 10.1073/pnas.0409713102 15716361 PMC552938

[B17] Zeng BY Dass B Owen A Rose S Cannizzaro C Tel BC Jenner P 6-hydroxydopamine lesioning differentially affects **α**-synuclein mRNA expression in the nucleus accumbens, striatum and substantia nigra of adult rats Neurosci Lett 2002 322 33 36 10.1016/S0304-3940(02)00083-6 11958837

[B18] Tanaka S Kondo H Kanda K Ashino T Nakamachi T Sekikawa K Iwakura Y Shioda S Numazawa S Yoshida T Involvement of interleukin-1 in lipopolysaccaride-induced microglial activation and learning and memory deficits J Neurosci Res 2011 89 506 514 10.1002/jnr.22582 21290410

[B19] Horai R Asano M Sudo K Kanuka H Suzuki M Nishihara M Takahashi M Iwakura Y Production of mice deficient in genes for interleukin (IL)-1α, IL-1β, IL1 α/β, and IL-1 receptor antagonist shows that IL-1β is crucial in turpentine-induced fever development and glucocorticoid secretion J Exp Med 1998 187 1463 1475 10.1084/jem.187.9.1463 9565638 PMC2212263

[B20] Tagawa Y Sekikawa K Iwakura Y Suppression of concanavalin A-induced hepatitis in IFN-gamma(-/-) mice, but not in TNF-alpha(-/-) mice: role for IFN-gamma in activating apoptosis of hepatocytes J Immunol 1997 159 1418 1428 9233639

[B21] Ohtaki H Funahashi H Dohi K Oguro T Horai R Asano M Owakura Y Yin L Matsunaga M Goto N Shioda S Suppression of oxidative neuronal damage after transient middle cerebral artery occlusion in mice lacking interleukin-1 Neurosci Res 2003 45 313 324 10.1016/S0168-0102(02)00238-9 12631467

[B22] Schmued LC Albertson C Slikker W Jr Fluoro-Jade: a novel fluorochrome for the sensitive and reliable histochemical localization of neuronal degeneration Brain Res 1997 751 37 46 10.1016/S0006-8993(96)01387-X 9098566

[B23] Glowinski J Iversen LL Regional studies of catechol-amines in the rat brain. I. The disposition of [3H[norepinephrine, [3H]dopamine and [3H]dopa in various regions of the brain. J Neurochem 1966 13 655 669 10.1111/j.1471-4159.1966.tb09873.x 5950056

[B24] Lindqvist D Kaufman E Brundin L Hall S Surova Y Hansson O Non-motor symptoms in patients with Parkinson’s **disease – correlations** with inflammatory cytokines in serum PLos One 2012 7 e47387 10.1371/journal.pone.0047387 23082161 PMC3474801

[B25] Boka G Anglade P Wallach D Javoy-Agid F Agid Y Hirsch EC Immunocytochemical analysis of tumor necrosis factor and its receptors in Parkinson’s disease Neurosci Lett 1994 172 151 154 10.1016/0304-3940(94)90684-X 8084523

[B26] Mogi M Harada M Kondo T Riederer P Inagaki H Minami M Nagatsu T Interleukin-1β, interleukin-6, epidermal growth factor and transforming growth factor-alpha are elevated in the brain from Parkinsonian patients Neurosci Lett 1994 180 147 150 10.1016/0304-3940(94)90508-8 7700568

[B27] Poot Godoy MC Tarelli R Ferrari CC Sarchi MI Pitossi FJ Central and systemic IL-1 exacerbates neurodegeneration and motor symptoms in a model of Parkinson’s disease Brain 2008 131 1880 1894 10.1093/brain/awn101 18504291 PMC2442423

[B28] Koprich JB Reske-Nielsen C Mithal P Isacson O Neuroinflammation mediated by IL-1**β** increases susceptibility of dopamine neurons to degeneration in an animal model of Parkinson’s disease J Neuroinflamm 2008 5 8 10.1186/1742-2094-5-8 PMC2292163 18304357

[B29] Zhang W Wang T Pei Z Miller DS Wu X Block ML Wilson B Zhang W Zhou Y Hong JS Zhang J Aggregated α-synuclein activates microglia: a process leading to disease progression in Parkinson’s disease FASEB J 2005 19 533 542 10.1096/fj.04-2751com 15791003

[B30] Li P Kaur C Lu J Sivakumar V Dheen ST Ling EA Expression of cyclooxygenase-2 and microsomal prostaglandin-E synthase in amoeboid microglial cells in the developing brain and effects of cyclooxygenase-2 neutralization on BV-2 microglial cells J Neurosci Res 2010 88 1577 1594 20025057 10.1002/jnr.22319

[B31] Jenner P Oxidative stress in Parkinson’s disease Ann Neurol 2003 53 S26 S38 10.1002/ana.10483 12666096

[B32] Gao HM Zhou H Hong JS NADPH oxidases: novel therapeutic targets for neurodegenerative diseases Trends in Pharmacol Sci 2012 33 295 303 10.1016/j.tips.2012.03.008 22503440 PMC3477578

[B33] Jenner P Olanow CW Oxidative stress and the pathogenesis of Parkinson’s disease Neurology 1996 47 S161 S170 10.1212/WNL.47.6_Suppl_3.161S 8959985

[B34] Izumi Y Sawada H Yamamoto N Kume T Katsuki H Shimohama S Akaike A Iron accelerates the conversion of dopamine-oxidized intermediates into melanin and provides protection in SH-SY5Y cells J Neurosci Res 2005 82 126 137 10.1002/jnr.20595 16108071

[B35] Sofic E Paulus W Jellinger K Riederer P Youdim MB Selective increase of iron in substantia nigra zona compacta of Parkinsonian brains J Neurochem 1991 56 978 982 10.1111/j.1471-4159.1991.tb02017.x 1704426

[B36] Lovell MA Ehmann WD Mattson MP Markesbery WR Elevated 4-hydroxynonenal in ventricular fluid in Alzheimer’s disease Neurobiol Aging 1997 18 457 461 10.1016/S0197-4580(97)00108-5 9390770

[B37] Fujita K Seike T Yutsudo N Ohno M Yamada H Yamaguchi H Sakumi K Yamakawa Y Kido MA Takaki A Katafuchi T Tanaka Y Nakabeppu Y Noda M Hydrogen in drinking water reduces dopaminergic neuronal loss in the 1-methyl-4-phenyl-1236-tetrahydropyridine mouse model of Parkinson’s disease PLoS One 2009 4 e7247 10.1371/journal.pone.0007247 19789628 PMC2747267

[B38] Liu W Kato M Akhand AA Hayakawa A Suzuki H Miyata T Kurokawa K Hotta Y Ishikawa N Nakashima I 4-hydroxynonenal induces a cellular redox, status-related activation of the caspase cascade for apoptotic cell death J Cell Sci 2000 113 635 641 10652256 10.1242/jcs.113.4.635

[B39] Ding M St Pierre BA Parkinson JF Medberry P Wong JL Rogers NE Ignarro LJ Merrill JE Inducible nitric-oxide synthase and nitric oxide production in human fetal astrocytes and microglia J Biol Chem 1997 272 11327 11335 10.1074/jbc.272.17.11327 9111039

[B40] Yamada T McGeer PL McGeer EG Lewy bodies in Parkinson’s disease are recognized by antibodies to complement proteins Acta Neuropathol 1992 84 100 104 10.1007/BF00427222 1502878

[B41] Choi DY Liu M Hunter RL Cass WA Pandya JD Sullivan PG Shin EJ Kim HC Gash DM Bing G Striatal neuroinflammation promotes Parkinsonism in rats PLos One 2009 4 e5482 10.1371/journal.pone.0005482 19424495 PMC2674956

[B42] Mizushima H Zhou CJ Dohi K Horai R Asano M Iwakura Y Hirabayashi T Arata S Nakajo S Takaki A Ohtaki H Shioda S Reduced postischemic apoptosis in the hippocampus of mice deficient in interleukin-1 J Comp Neurol 2002 448 203 216 10.1002/cne.10262 12012430

[B43] Sato A Ohtaki H Tsumuraya T Song D Ohara K Asano M Iwakura Y Atsumi T Shioda S Interleukin-1 participates in the classical and alternative activation of microglia/macrophages after spinal cord injury J Neuroinflamm 2012 9 65 10.1186/1742-2094-9-65 PMC3353190 22483094

